# MEK1/2 inhibitor ATR-002 reshapes host transcriptome and modulates immune regulatory genes in SARS-CoV-2 infection

**DOI:** 10.3389/fimmu.2026.1724353

**Published:** 2026-05-29

**Authors:** Franziska Rodner, Klaus Schughart, Stephan Ludwig, André Schreiber

**Affiliations:** 1 Institute of Virology Muenster, University of Muenster, Muenster, Germany; 2 Department of Microbiology, Immunology and Biochemistry, University of Tennessee Health Science Centre, Memphis, TN, United States

**Keywords:** immune response, inhibitor, MEK, SARS-CoV-2, transcriptome, virus

## Abstract

**Introduction:**

SARS-CoV-2 portrays a public health threat because severe progression of infections can lead to hospitalization. Severity is often characterized by a systemic inflammatory response in later stages, along with disbalance in cytokine production, multi-organ failure, or acute respiratory distress syndrome (ARDS). As severe infections can be challenging due to limited treatment windows for direct-acting antivirals, host-targets, e.g., the Raf/MEK/ERK signaling cascade, came into focus. Specifically, MEK1/2 has been suggested as a target for antiviral and anti-inflammatory therapy. A recent phase II clinical trial showed the efficacy of the MEK1/2 inhibitor zapnometinib (ZMN/ATR-002) in hospitalized COVID-19 patients. The anti-inflammatory action of ATR-002 was only studied on some candidate cytokines.

**Methods:**

To generate a comprehensive overview of transcriptional effects on immune regulatory genes, we performed a transcriptome analysis of SARS-CoV-2-infected Calu-3 cells in the presence or absence of ATR-002.

**Results:**

Gene expression data revealed significant upregulation of innate immune-response-related genes, e.g., cytokines (*CXCL10* and *CCL5*), which are associated with severe cases of COVID-19, or ISGs (*MX1* and *OASL*) induced by the infection. ATR-002 could reverse this effect in the infection. To exclude the possibility that regulated immune-response-related genes are not affected by the reduced viral load upon inhibitor treatment, but by the pathway interference itself, MEK1/2 inhibition in a non-infectious scenario was tested. Lowered immune regulatory gene expression was traced back to impaired viral replication, as shown by different genes affected in the non-infected contrast. However, some target genes (EGR1, IFNL1, and NLRP1), which could be connected to transcriptional regulation via the Raf/MEK/ERK axis, proved to be involved in the modulation of innate immunity. Especially NLRP1 expression was newly identified as a target of MEK1/2 inhibition, which links Raf/MEK/ERK signaling to caspase-1 activity and the subsequent processing of IL-1β, a major driver of hyperinflammation. MEK1/2-inhibition-mediated regulation of other targets, such as EGR1, may exert additional immune regulatory processes, influencing inflammation in SARS-CoV-2 infections.

**Discussion:**

With our analysis of transcriptional regulation after SARS-CoV-2 infection and the potential influence of ATR-002, we were able to identify ERK1/2-dependent gene expression and candidate genes (EGR1, IFNL1, and NLRP1), which could be involved in the regulation of innate immune-response-related processes.

## 1 Introduction

Respiratory viral infections, can have severe consequences for patients, as shown by the most recent outbreak of the SARS-CoV-2 pandemic, which caused over 7 million deaths (1). The driving force in the development of severe complications in COVID-19 patients, such as dyspnea, hypoxemia, and sepsis that can result in multi-organ damage and acute respiratory distress syndrome (ARDS), is a severe systemic inflammatory response characterized by the dysregulation of cytokine expression (2, 3). After viral entry, SARS-CoV-2 infection induces the expression of pro-inflammatory cytokines and interferons (IFNs). The recognition of viral RNAs by pattern recognition receptors (PRRs) activates distinguished signaling pathways, resulting in the activation of different transcription factors such as NF-κB and IRF. Whereas the inflammatory response in mild cases can diminish viral load in an adequate time and returns rapidly to basal levels, severe cases show a prolonged expression window of pro-inflammatory cytokines and lower IFN expression, leading to delayed viral clearance (4). In accordance with these findings, elevated levels of several pro-inflammatory cytokines and chemokines (IL6, IL8, IL10, TNFα, IP10, CCL2, and CCL5), NLRP3 inflammasome activation as well as IL-18 and IL-1β secretion, known as a cytokine storm, were observed *in vitro*, *in vivo*, and in patients (5–9). The resulting recruitment of immune cells such as T cells and monocytes to the lung causes the maintenance of the inflammatory response and the progression of the disease (10).

Early in SARS-CoV-2 infections, direct-acting antivirals (DAAs) are the therapeutics of choice aiming to reduce the viral load in infected individuals. The WHO recommends Paxlovid, consisting of nirmatrelvir and ritonavir, which inhibits the viral 3CLpro protease, in patients with non-severe COVID-19 at high or moderate risk of hospitalization (11, 12). Remdesivir and molnupiravir treatments, both nucleoside analogues, are only conditionally recommended in non-severe COVID-19 patients at high risk of hospitalization (11). However, in critically ill late-stage patients, these compounds are not effective, and diseased individuals are rather treated in regard to their dysbalanced immune response to diminish the elevated expression levels of pro-inflammatory cytokines using anti-inflammatory drugs. For those patients with severe or critical COVID-19, the WHO advises a combinational treatment of systemic corticosteroids (dexamethasone), IL6 receptor blocker (tocilizumab or sarilumab), and Janus kinase (JAK) inhibitors (baricitinib, ruxolitinib, and tofacitinib) (11, 12). To combat such severe infections most efficiently, a well-timed treatment is indispensable. DAAs often lack anti-inflammatory properties. Nevertheless, switching too early in the course of the infection to anti-inflammatory drugs may hinder viral clearance. In addition, a combinational therapy of corticosteroids and DAAs is not advisable due to possible drug interferences and side effects (13). To overcome such problems in a sensitive medication plan, host-targeted antivirals (HTAs) could be promising alternatives. HTAs target host-specific processes, known to be relevant for efficient viral replication and may offer a secondary benefit by downregulating inflammatory responses, potentially reducing the risk of cytokine storm induction (14, 15). In addition, they have the potential to act in a widespread way by interfering with the host cell machinery, thereby covering a broad spectrum of viruses and posing a lower risk of drug resistance selection (16).

In this study, we focused on the impact of the specific MEK1/2 inhibitor ATR-002, also known as zapnometinib (ZMN), on the SARS-CoV-2-induced pro-inflammatory cytokine response. It was shown that various viruses, like SARS-CoV-2, exploit this pathway to promote replication, and the inhibition of MEK1/2 interferes with the entry mechanism and is regulating pro-inflammatory cytokine expression as well (17–23). Based on these observations, we wanted to further elucidate the link of the distinctive Raf/MEK/ERK pathway and innate immune-response-related mechanisms on the transcriptional level. So far, the impact of the pathway has only been shown on selected candidate immune-related genes. The current study is a transcriptome-wide extension to our previously published findings and aimed to generate a more comprehensive analysis on how MEK1/2 inhibition affects the virus-induced host gene expression profile in SARS-CoV-2-infected epithelial cells, especially with a focus on potential changes in the gene expression of innate immune-response-related genes, such as pro-inflammatory cytokines or ISGs, and potential underlying molecular mechanistic processes.

## 2 Materials and methods

### 2.1 Infection and inhibitor treatment of Calu-3 cells

Calu-3 cells were grown in Dulbecco’s modified Eagle’s medium (Gibco, Waltham, MA, USA) containing 10% fetal bovine serum (FBS) and 1% penicillin/streptomycin (P/S) at 37°C and 5% CO_2_. The human airway epithelial cell line (Calu-3) presented in this study, obtained from ATCC (HTB-55), were taken from the collection of the Institute of Virology Muenster. Cells were grown to confluency and infected with SARS-CoV-2 D614G (hCoV-19/Germany/FI1103201/2020) or SARS-CoV-2 Omicron B.1.1.529 (BE.1.1) (hCoV-19/Germany/NW-IFH-359092/2022) (MOI 0.01) using DMEM/HAM (Gibco) infection media (1% sodium pyruvate, 1% P/S, 1% non-essential amino acids, 1% HEPES, 2% FBS). At 1 h after infection, ATR-002, also known as zapnometinib (ZMN) (Atriva Therapeutics, Tübingen, Germany) treatment was initialized using concentrations of 1, 10, and 50 µM in DMEM/HAM. The solvent DMSO (0.1%) served as negative control. At 48 h post-infection (h.p.i.), RNA-seq was performed. Each condition was prepared in biological triplicates. All experiments were performed under biosafety level 3 conditions.

### 2.2 RNA isolation and RNA sequencing

RNA isolation was performed with the RNeasy Mini Plus kit (Qiagen, Hilden, Germany) according to the manufacturer’s protocol. RNA from lysed and homogenized cells was purified by using a column-based system and washed three times before elution with 30 µL RNase-free ddH_2_O. The samples were stored at -80 °C until further processing.

The RNA quality and integrity of total RNA were controlled using Agilent Tape Station 4200, D1000 Tape (Agilent Technologies, Waldbronn, Germany). Poly^+^RNA was purified from 1 µg total RNA using NEBNext Sample Purification Beads (E7666L, New England Biolabs). The RNA sequencing library was prepared with Illumina Stranded mRNA Prep (Illumina, San Diego, CA, USA) and IDT for Illumina DNA/RNA UD Indexes Set A (Illumina). The purified samples were sequenced in Illumina NovaSeq 6000 with the NovaSeq 6000S2 reagent kit (338 cycles, paired end 2 × 159 bp) with an average read of 51.8 M reads per RNA sample. Sequencing was performed by the Core Facility Genomics of the Medical Faculty Münster.

### 2.3 cDNA synthesis and qRT-PCR

In order to perform qRT-PCR for the validation of RNA-seq data, previously isolated RNA was converted into cDNA via reverse transcription using RevertAid First Strand cDNA-Synthesis-Kit (Thermo Fisher Scientific, Waltham). According to the manufacturer’s protocol, 1 µg of template RNA was mixed on ice with 0.5 µg of Oligo dt Primer (16mer) and supplemented with RNase-free ddH_2_O. The samples were incubated for 5 min at 70°C, followed by incubation on ice for 1 min and at 37°C for 2 min. A master mix of ReverseAid H minus Reverse Transcriptase (25 U final concentration) (Thermo Fisher Scientific), 5× Reaction Buffer (1× final concentration) 1 mM dNTP Mix, and ddH_2_O was prepared in a final volume of 8 µL per reaction. The master mix together with the annealed RNA was incubated for 10 min at 37°C, followed by 1-h incubation at 42°C, and finally for 2 min at 70°C. For the subsequent analysis of mRNA expression of IFNL1 (forward: 5′-AACTGGGAAGGGCTGCCACATT-3′, reverse: 5′-GGAAGACAGGAGAGCTGCAACT-3′), EGR1 (forward: 5′-AGGGAAAGGGGAAAGAAAGG-3′, reverse: 5′-AATTGGGGAAGGGGAAGTG-3′), GAPDH (forward: 5′-GCCCACTTGATTTTGGAGG-3′, reverse: 5′-GGCCATGACCAACAAGTGTCTCCTCC-3′), and NLRP1 (forward: 5′-ATTGAGGGCAGGCAGCACAGAT-3′, reverse: 5′-CTCCTTCAGGTTTCTGGTGACC-3′) using qRT-PCR, primer mix solutions consisting of 10 µM of each primer were prepared. 2× Brilliant II SYBR^®^ Green QPCR Master Mix (4 µL) (Agilent Technologies) was mixed with the respective primer solutions and ddH_2_O to a final volume of 4.8 µL and combined with 7.2 µL of diluted cDNA. The measurement was performed with LightCycler 480II (Roche, Basel, Switzerland) using one cycle for 3 min at 95°C, 50 cycles of 5 s at 95°C, followed by 10 s at 60°C, and a final melting curve for 10 min ramping from 55 °C to 95°C. Analysis was performed according to the ΔΔCT method (24).

### 2.4 Western blot analysis

The protein expression of cellular proteins was measured via SDS-PAGE and Western blot analysis. Total cell lysates were collected by lysis in radio-immunoprecipitation buffer (RIPA) (1 mM EDTA (pH 8.0) (Roth, Karlsruhe, Germany), 10% (v/v) glycerol (Roth), 137 mM NaCl (Roth), 1% (v/v) NP-40 (Sigma-Aldrich, Munich, Germany), 0.1% (w/v) SDS (Roth), 0.5% (w/v) sodium deoxycholate (Serva), and 25 mM Tris-HCl (pH 8.0) (Roth)) and protease inhibitors, which were supplemented after (5 µg/mL aprotinin (1:1,000) (Roth), 5 µg/mL leupeptin (1:1,000) (Serva, Heidelberg, Germany), 0.3 mM Pefablock (1:1,000) (Roth), 1 mM Sodium orthovanadate (1:100) (Sigma-Aldrich), 5 mM Benzamidine (1:200) (Sigma-Aldrich)). The lysates were centrifuged for 10 min at 20817 rcf and 4°C and afterward denatured with 5× Leammli buffer for 10 min at 95°C. The proteins were separated by SDS-PAGE and subsequently transferred to nitrocellulose membranes via western blotting. The membranes were blocked in 5% BSA (w/v) (Roth) in TBS-T buffer (pH 7.6) (3 M NaCl (Roth), 1 M Tris (Roth), and 0.2% (v/v) Tween20 (Roth)) for 1 h at RT. Primary antibodies (**Supplementary Table 1**) (1:1,000 diluted in 5% BSA in TBS-T) were incubated overnight at 4°C (**Supplementary Table 1**). Secondary antibodies (1:3,000 diluted in TBS-T) were incubated for 1 h at RT. Chemiluminescence was measured with the Li-CorOdissey® Fc Imaging System and further analyzed by using the Image Studio^TM^ (LiCor) software.

### 2.5 Cell cytotoxicity assay

Calu-3 cells were seeded in 96-well flat-bottom cell culture plates and, at 48 h later, treated with different concentrations of ATR-002 (1.56, 3.125, 6.25, 12.5, 25, 50, and 100 µM) for 48 h further. Then, 15 µL of sterile water or 10% Triton-X solution (CytoSelect™ LDH Cytotoxicity Assay Kit) (Cell Biolabs Inc.) was added to the cells, and these were incubated for 10 min at room temperature. In a clear cell culture plate, 10 µL of LDH cytotoxicity assay reagent (CytoSelect™ LDH Cytotoxicity Assay Kit) was mixed with 90 µL of supernatant. The samples were incubated for 30 min at 37°C and 5% CO_2_. Measurement was performed using Tristare 3 Multimode Reader (Berthold Technologies, Bad Wildbad, Germany) at 450 nm.

### 2.6 Bioinformatic analysis

Reads from RNA sequencing (RNA-seq) were quality-checked with FastQC (version 0.11.9) and trimmed using Trimgalore (version 0.6.7, https://www.bioinformatics.babraham.ac.uk/projects/trim_galore/) with default settings. The trimmed reads were mapped to human genome annotation GRCh38 (Homo_sapiens.GRCh38.111.gtf, release 111) via STAR aligner (Dobin and Gingeras, 2015) with default settings. Further analysis and visualization of expression data were conducted using the R software (version 4.2.1, and 4.4.3 (R_Core_Team, 2013a) and RStudio (versions 2022.07.2 and 2024.12.1 (RStudio)). The mapped reads were counted at the gene level via RsubRead (version 1.32.4, (Liao et al., 2019)). Annotation of human genes was performed using package biomaRt (version 2.52.0, assessed 120325 (Durinck et al., 2005)). Raw counts for host genes were normalized and log_2_-transformed by function rlogTransformation from the DESeq2 package (version DESeq2_1.46.0 (25). An increment was added to the normalized values to make all values positive. Virus reads were identified by mapping to the virus genome (hCoV-19GermanyNW-IMVUM_FI11032012020, GISAID database ID: EPI_ISL_463008) using the same protocol as above. Raw virus reads were then normalized as counts per million (CPM). For identification of differentially expressed genes (DEGs), package DESeq2 was used with the following model: design = ~ donor + group; groups: “uninf_untrt_48h”, “uninf_DMSO_48h”, “uninf_ATR1uM_48h”, “uninf_ATR10uM_48h”, “uninf_ATR50uM_48h”, “uninf_ATR100uM_48h”, “SARS_untrt_48h”, “SARS_DMSO_48h”, “SARS_ATR1uM_48h”, “SARS_ATR10uM_48h”, “SARS_ATR50uM_48h”, and “SARS_ATR100uM_48h”. DEGs were identified using a threshold for the adjusted *p*-value of <0.05 and more than a twofold (|log2| > 1) difference in expression levels. Volcano plots were generated with the package EnhancedVolcano, version 1.14.0 (26) Heatmaps were generated with the function heatmap2 of package gplots (version 3.1.3; https://github.com/talgalili/gplots).

Functional analyses of DEGs were performed using the functional enrichment analysis web tool Webgestalt 2019 (https://2019.webgestalt.org). The method of over-representation analysis (ORA) was used to assign non-redundant gene ontology terms of biological processes (GO-BP). As parameters for the enrichment analysis, a minimum number of five IDs per category had to be assigned. The cutoff criteria for further analysis was the 10 strongest regulated categories according to their false discovery rate (FDR) ≤0.05. The data shown represent the weighted set cover to reduce the redundancy of the categories. Overlapping gene sets were analyzed with the web tool of the University of Gent (Bioinformatics and Evolutionary Genomics (https://bioinformatics.psb.ugent.be/webtools/Venn/)).

## 3 Results

### 3.1 MEK1/2 inhibition reduces viral load upon SARS-CoV-2 infection

We have shown previously that MEK1/2 inhibition leads to reduced virus replication in SARS-CoV-2 and influenza A virus infections *in vitro* and *in vivo* (17, 18, 27). First, we aimed to confirm these findings in the infection setting that we used for our transcriptomic analysis.

**Figure 1:**
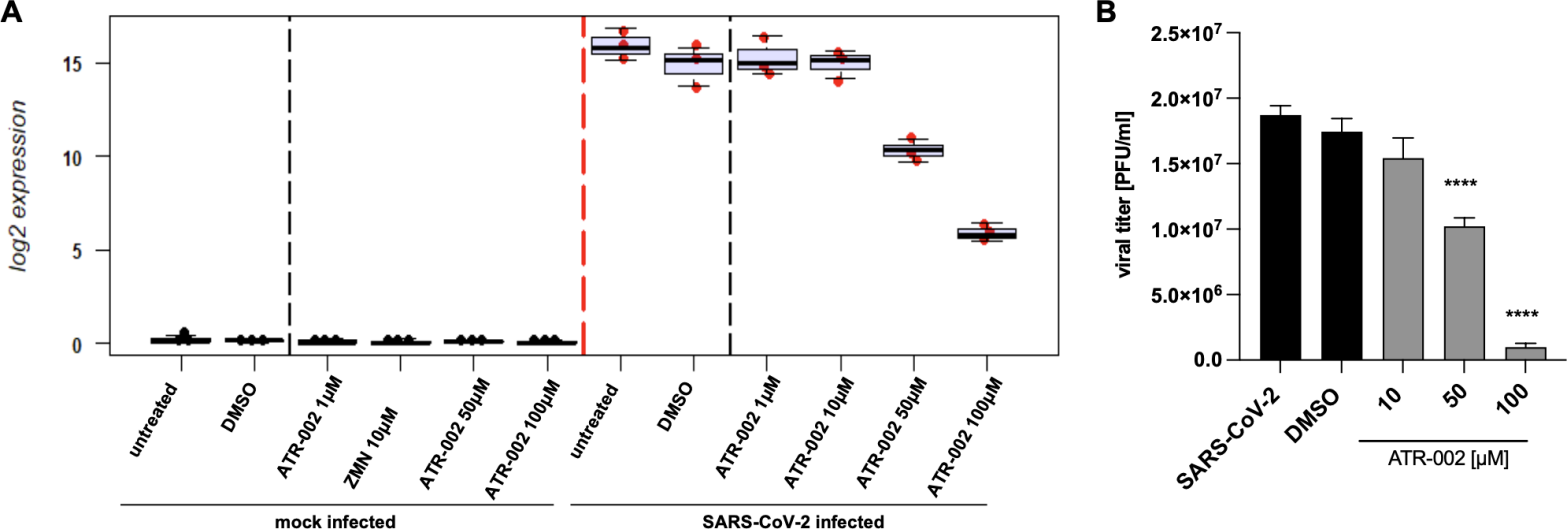
Antiviral effect of ATR-002 against SARS-CoV-2. Viral RNA expression and viral titers after 48 hpi with SARS-CoV-2 (D614G-FI) (MOI 0.01) in Calu-3 cells after MEK1/2 inhibition with the inhibitor ATR-002. Mock or SARS-CoV-2-infected Calu-3 cells were treated with different concentrations of ATR-002 (1, 10, 50, and 100 μM). Untreated and DMSO (0.1%)-treated cells served as negative controls. The data represent three biological replicates. **(A)** CPM (log_2_ values of normalized expression levels) of summed viral gene expression mapped to the viral genome. **(B)** Viral titers in PFU/mL of ATR-002-treated SARS-CoV-2 infection (10, 50, and 100 μM). The data passed an ordinary one-way ANOVA followed by Dunnett’s multiple comparison test (****p ≤ 0.0001).

Calu-3 cells were either non-treated, DMSO-treated, or ATR-002-treated and were infected with SARS-CoV-2 for 48 h (or left uninfected (mock)). To ensure successful infection and sufficient blockade of viral replication due to ATR-002 inhibitor treatment, the expression of viral RNAs and viral titers was assessed after 48 h (**Figure 1**). The expression of viral RNAs (**Figure 1A**) was not affected in SARS-CoV-2-infected control-treated cells, while inhibition with ATR-002 led to a dose-dependent reduction. Similarly, viral titers showed a decrease of progeny viral particles after inhibition of MEK1/2. The antiviral effects were dose dependent, with a significant reduction to 58.68% ± SD 4.53 already at 50 µM ATR-002 (**Figure 1B**), showing that the experiments were conducted under inhibitory conditions. The concentrations of the inhibitor used were below the calculated CC_50_ values reported by Schreiber et al. (2022) (17), which were additionally confirmed via testing of cell viability (**Supplementary Figure 1**). Additionally, a detailed transcriptome analysis was performed for samples in which Calu-3 cells were either mock or SARS-CoV-2-infected and treated with DMSO or 50 µM of the inhibitor ATR-002.

### 3.2 MEK1/2 inhibition results in altered mRNA expression profiles of SARS-CoV-2-infected Calu-3 cells

**Figure 2:**
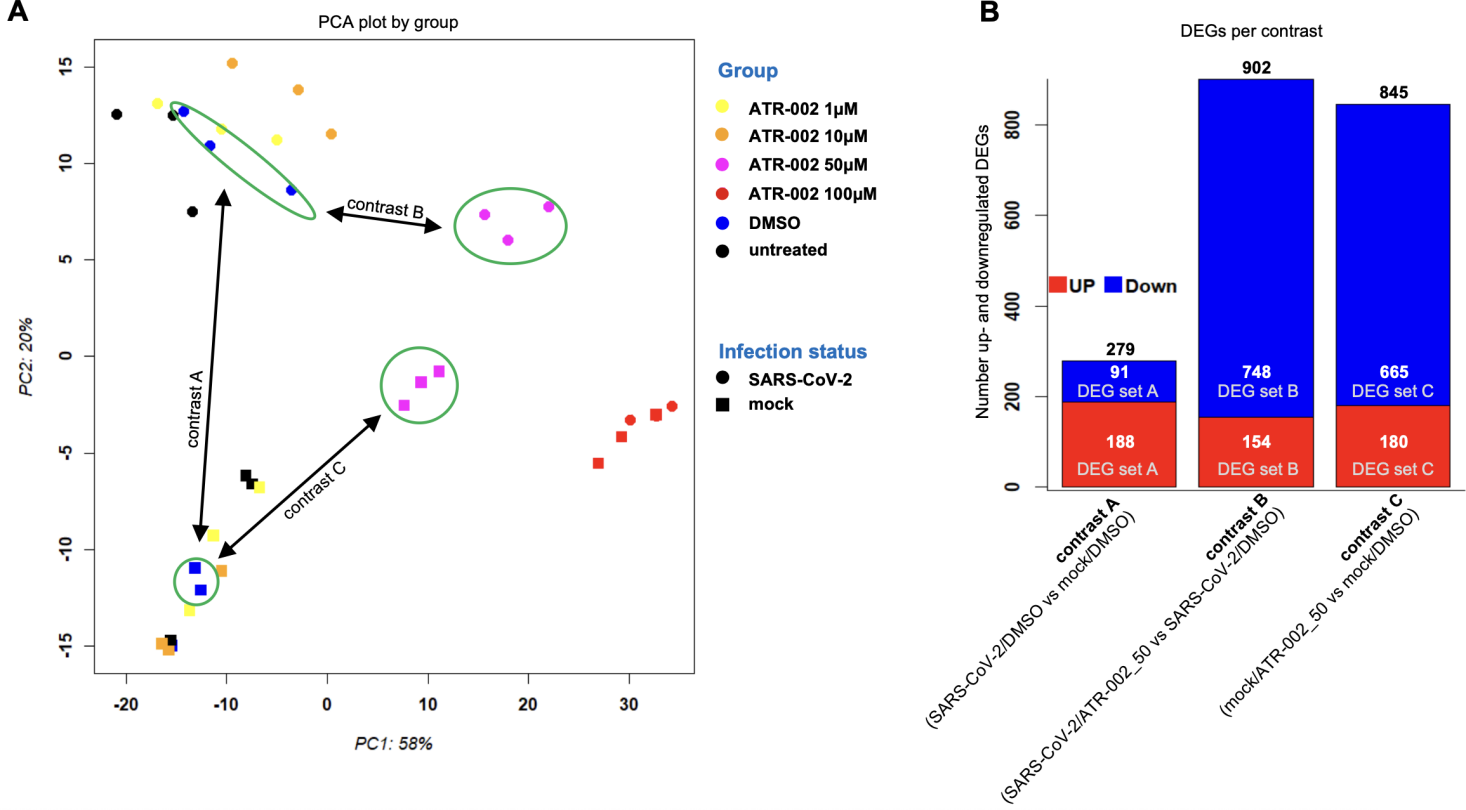
Transcriptional shift induced by ATR-002 in SARS-CoV-2 infection. Analysis of mRNA expression profiles of mock and SARS-CoV-2-infected Calu-3 cells treated with different concentrations of ATR-002 (1, 10, 50, and 100 μM) after 48 hpi. **(A)** Principal component analysis (PCA) representing PC1 and PC2 of normalized transcriptome expression values of all 12 experimental groups. Each dot represents an independent biological replicate. Four experimental groups (mock/DMSO, mock/ATR-002_50, SARS-CoV-2/DMSO, and SARS-CoV-2/ATR-002_50) were selected for a more detailed analysis (green circles). **(B)** Total number of up- (red) and downregulated (blue) DEGs of contrast A (SARS-CoV-2/DMSO vs. mock/DMSO), contrast B (SARS-CoV-2/ATR-002_50 vs. SARS-CoV-2/DMSO), and contrast C (mock/ATR-002_50 vs. mock/DMSO). DEG set A (DOWN) represents the downregulated DEG set of contrast A, whereas DEG set A (UP) represents the upregulated DEG set of the same contrast. The same nomenclature was used for the DEG contrasts B and C.

To gather a first overview on the overall changes in the SARS-CoV-2-induced transcriptome after MEK1/2 inhibition, a principal component analysis (PCA) was performed. A clear separation of mock and SARS-CoV-2-infected samples as well as clustering of inhibitor-treated samples could be demonstrated. Inhibition of MEK1/2 at a concentration of 50 µM during viral infection further enhanced the separation between mock-infected and SARS-CoV-2-infected samples (**Figure 2A**), indicating that the virus-induced mRNA expression profile is further shifted after treatment with 50 µM ATR-002. Therefore, this concentration was considered for further analyses. First, DMSO-specific effects were excluded by comparing SARS-CoV-2-infected + DMSO-treated (SARS-CoV-2/DMSO) with SARS-CoV-2 infected + untreated (SARS-CoV-2/untreated) samples (**Supplementary Table 2**). No significant changes in gene expression were detected, indicating that DMSO, as a solvent, did not induce transcriptional changes. For the analysis of differentially expressed genes (DEGs), three contrasts were chosen for further processing. To identify SARS-CoV-2-infection-specific induced alterations in gene expression, SARS-CoV-2 infection + DMSO treatment were compared to mock-infected + DMSO-treated Calu-3 cells (contrast A = SARS-CoV-2/DMSO vs. mock/DMSO), revealing 91 down- and 188 upregulated genes, respectively (**Figure 2B**). To identify the effects of ATR-002 in a SARS-CoV-2 infection scenario on gene regulation, SARS-CoV-2-infected + ATR-002-treated (50 µM) samples were compared to SARS-CoV-2-infected + DMSO-treated samples (contrast B = SARS-CoV-2/ATR-002_50 vs. SARS-CoV-2/DMSO). Here 748 genes were downregulated and 154 genes were upregulated (**Figure 2B**). Lastly, ATR-002-inhibitor-induced changes in gene expression were shown by a contrast of mock-infected + ATR-002-treated (50 µM) with mock-infected + DMSO-treated samples (contrast C = mock/ATR-002_50 vs. mock/DMSO). ATR-002 treatment resulted in 665 downregulated genes and led to the upregulation of 180 genes at 48 h.p.i. (**Figure 2B**).

### 3.3 SARS-CoV-2 infection leads to an induced expression of innate immune-response-related genes in Calu-3 cells

**Figure 3:**
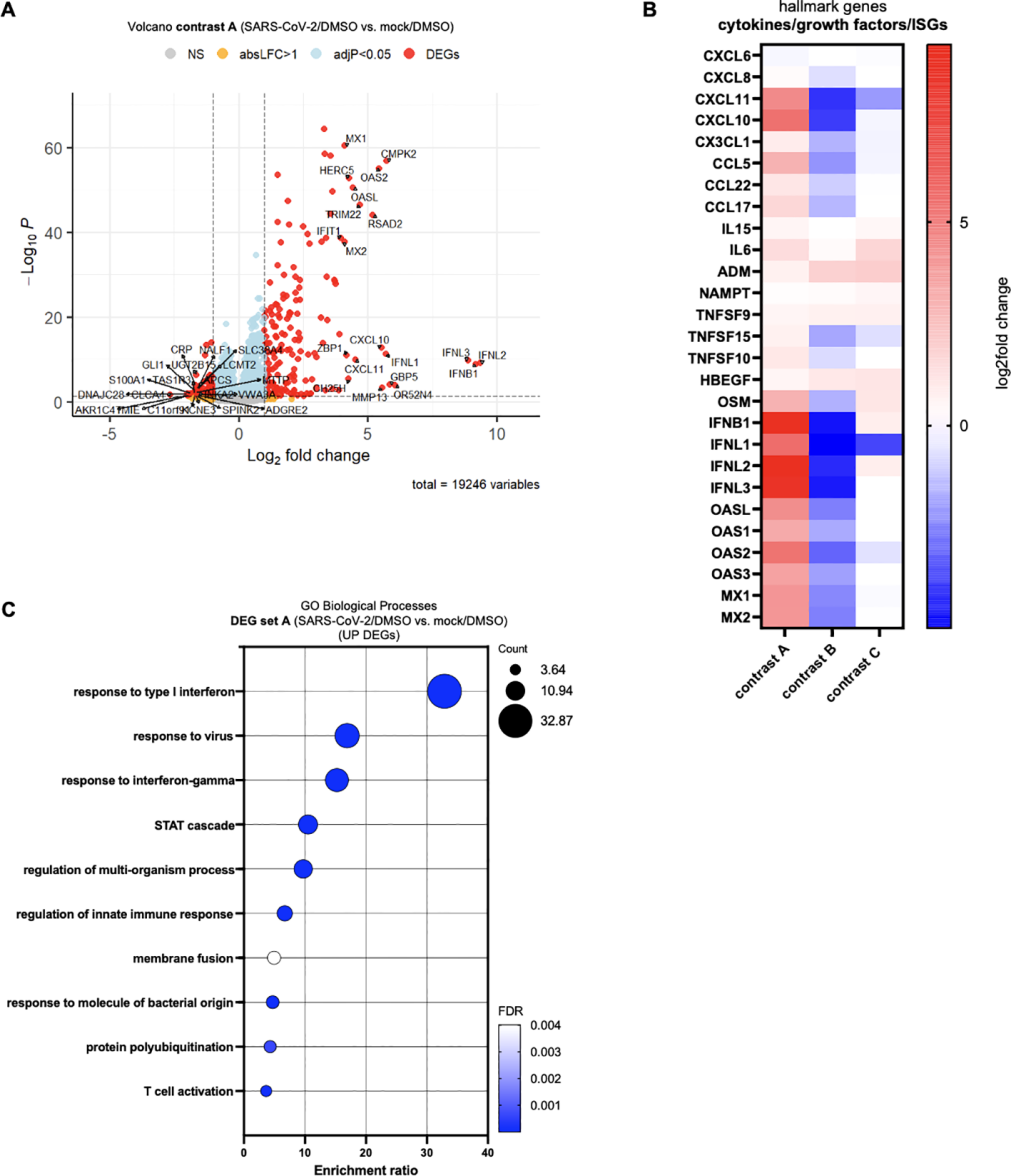
Overview of DEGs in SARS-CoV-2-infected Calu-3 cells (contrast A: SARS-CoV-2/DMSO vs. mock/DMSO). **(A)** Volcano plot of contrast A showing all significant DEGs (red) and annotation (gene symbols) of the top 20 differentially expressed genes (up- and downregulated). Genes with adj. *p*-value <0.05 are shown in blue. Genes with an absolute log_2_ fold change >1 are shown in yellow. Log_2_ fold change is plotted on the x-axis and – log_10_ adj. *p*-value is plotted on the y-axis. **(B)** Heatmap of hallmark cytokines, growth factors in inflammation, and ISGs comparing log_2_ fold changes of DEG set A (UP) and DEG set B and C (DOWN). **(C)** Over-representation analysis (ORA) of DEG set A showing the gene ontology terms of biological processes (GO-BPs) of upregulated DEGs. The circle size corresponds to the overlapping counts of genes with the respective gene set.

It is a common observation that virus infections initiate a cellular defense program that leads to changes in mRNA expression, resulting, e.g., in innate immune gene activity. The investigated SARS-CoV-2 infection induced a total number of 279 DEGs (**Figure 2B**). Upregulation of 188 DEGs was observed in contrast A, where the expression levels in infected samples shifted in contrast to the basal expression of mock-infected Calu-3 cells (**Supplementary Figure 2A**). A volcano plot depicts the distribution of all DEGs as well as the gene identifier of the top 20 strongest downregulated and upregulated genes by log_2_ fold change (>1) (**Figure 3A**). Among the strongest upregulated genes were cytokines (*CXCL10* and *CXCL11*), interferons (*IFNB1* and *IFNL1-3*), and interferon -stimulated genes (ISGs) (*OASL* and *MX1*) (**Supplementary Tables 3**, **4**). Other pro-inflammatory cytokines as described in the “Introduction”, which are dysregulated in severe cases of SARS-CoV-2 infection and represent hallmark genes for inflammation according to MSigDB, e.g., *IL6* and *CCL5*, were upregulated as well (**Figure 3B**), confirming the robustness of our data.

To categorize all DEGs of contrast A in terms of their biological functions, gene enrichment analysis was performed using the web tool Webgestalt 2019. Gene ontology (GO) categories describing biological processes (GO-BP) were assigned to all up- and downregulated genes induced by SARS-CoV-2 infection. The method of over-representation analysis (ORA) identified categories primarily associated to the regulatory processes of the immune response, like response to type I interferons (GO:0034340) or response to virus (GO:0009615) (**Figure 3C**) for DEG set A (UP). Analyzing all downregulated genes (DEG set A (DOWN)) in terms of their biological function, we did not find significantly relevant categories with a false discovery rate (FDR) ≤0.05. It may be noteworthy that categories below the set threshold (FDR > 0.05) included genes related to, e.g., ventricular system development (GO:0021591) or double-strand break repair (GO:0006302), but no common category, like immune response as for DEG set A (UP), represented the downregulated genes (**Supplementary Figure 3**). As the low overlapping count of DEGs with the defined gene sets of the GO categories resulted in FDR >0.05, these categories and genes were not used for further analysis.

### 3.4 MEK1/2 inhibition in SARS-CoV-2 infection leads to the downregulation of immune-response-related genes in Calu-3 cells

**Figure 4:**
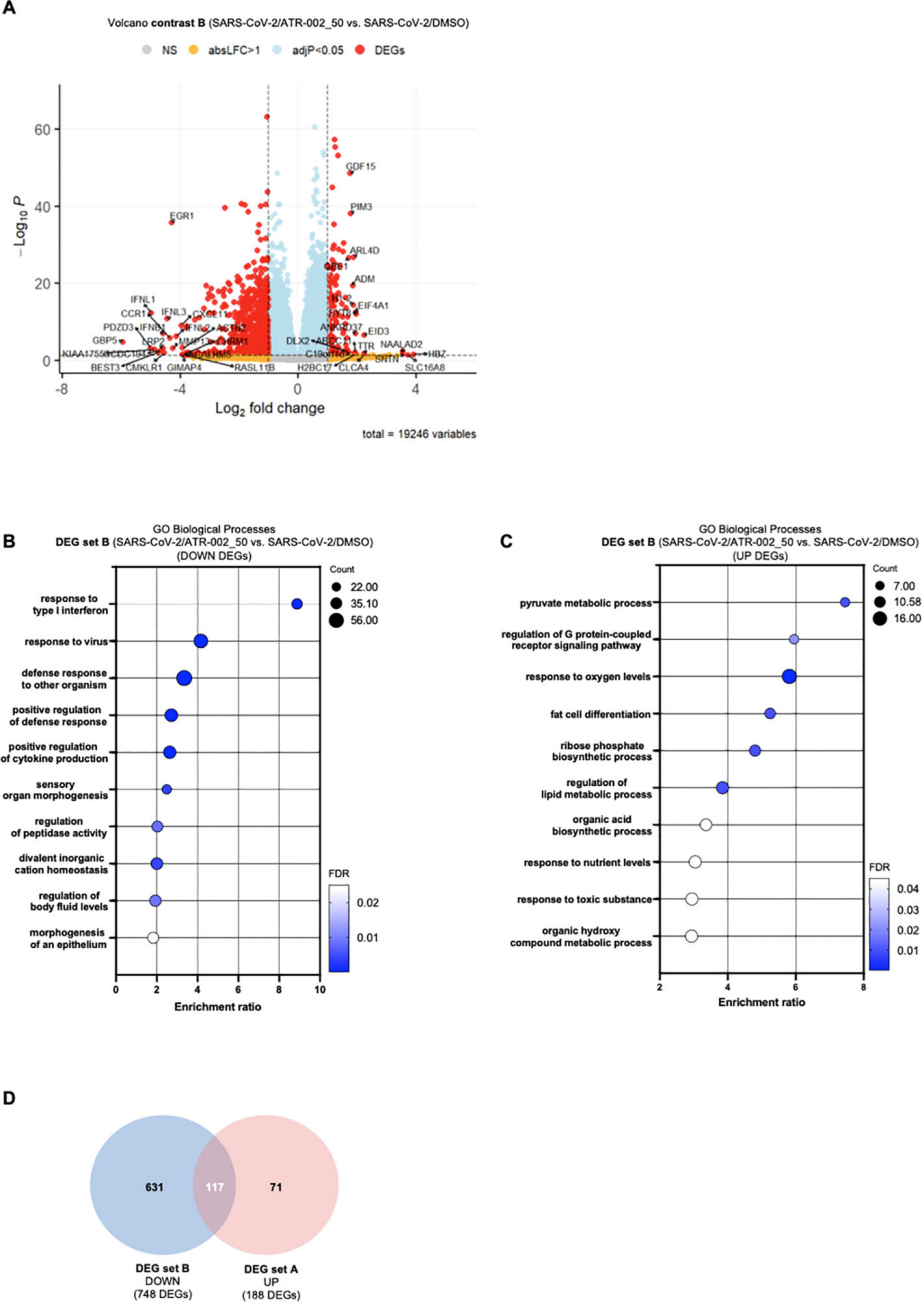
Overview of DEGs in ATR-002 (50 μM)-treated SARS-CoV-2-infected Calu-3 cells (contrast B: SARS-CoV-2/ATR-002_50 vs. SARS-CoV-2/DMSO). **(A)** Volcano plot of contrast B showing all significant DEGs (red) and annotation (gene symbols) of the top 20 differentially expressed genes (up- and downregulated). Genes with adj. *p*-value <0.05 are shown in blue. Genes with an absolute log_2_ fold change >1 are shown in yellow. Log_2_ fold change is plotted on the x-axis and –log_10_ adj. *p*-value is plotted on the y-axis. **(B)** Over-representation analysis (ORA) of DEG set B (DOWN) showing the gene ontology terms of biological processes (GO-BPs) of downregulated DEGs. **(C)** Over-representation analysis (ORA) of DEG set B (UP) showing the gene ontology terms of biological processes (GO-BPs) of upregulated DEGs. (B/C) The circle size corresponds to the overlapping counts of genes with the respective gene set. **(D)** Two-way Venn diagram of DEG set B (DOWN) and DEG set A (UP), showing the overlapping genes upregulated during SARS-CoV-2 infection and downregulated following ATR-002 treatment in SARS-CoV-2 infection.

In previous studies, the impact of MEK1/2 inhibitor treatment on cellular gene expression was only shown with some selected candidate genes. We thus aimed to analyze how MEK1/2 inhibition would affect the global cellular gene expression profile in the context of SARS-CoV-2 infection. Therefore, the gene expression profile of DMSO-treated infected cells was compared to infected cells treated with ATR-002 (50 µM) (contrast B). ATR-002 treatment in the SARS-CoV-2 infection induced 902 DEGs in total. The corresponding heatmap showed a clear shift of downregulated gene expression of a large proportion of DEGs (**Supplementary Figure 2B**) in the presence of the inhibitor. Considering the top 20 dysregulated genes for this contrast (contrast B) depicted as a volcano plot (**Figure 4A**; **Supplementary Tables 5**, **6**), strong downregulation by log_2_ fold change was observed for some interferon (e.g., *IFNL1* and *IFNL3*) and cytokine (e.g., *CXCL11*) gene transcripts (**Supplementary Table 5**). Among the cytokines associated with a severe outcome of SARS-CoV-2 infection, only *CCL5* and *CXCL10* were downregulated by the inhibitor treatment during the infection (**Figure 3B**).

ORA of GO-BPs from the DEG set B with 748 downregulated genes grouped those genes into 10 categories, with five of them associating to the regulatory processes of the immune response, e.g., response to type I interferon (GO:0034340) or response to virus (GO: 0009615) (**Figure 4B**), which were found both in DEG set A (UP) and DEG set B (DOWN). The remaining categories included diverse processes, e.g., regulation of body fluid levels (GO:0050878) or sensory organ morphogenesis (GO:0090596). Considering the upregulated genes induced by the ATR-002 treatment, GO analysis assigned many genes to categories involved in metabolic processes, e.g., pyruvate metabolic process (GO:0006090) or response to oxygen levels (GO:0070482) (**Figure 4C**). This phenotype might be explainable by metabolic compensation induced by cellular stress through the inhibition of MEK1/2 (28).

An increase in the expression of immune-response-related genes in SARS-CoV-2 infection was found by corresponding GO annotations for immune-response-related processes (**Figure 3C**). The same (response to type I interferon: GO:0034340, response to virus: GO:0009615) or similar GO-BP categories (defense response to other organism (GO:0098542)) appear for those genes downregulated in a MEK1/2-inhibitor-treated infection (DEG set B (DOWN)). To determine if the same genes involved in immune regulatory processes are affected in both DEG sets, an overlap of these two gene DEG sets was performed. Out of 748 genes downregulated by inhibitor treatment during infection (DEG set B (DOWN)) and 188 genes upregulated in SARS-CoV-2 infection (DEG set A (UP)), 117 genes (**Supplementary Table 7**) could be identified in both gene subsets (**Figure 4D**). The overlapping genes could also be categorized into immune-response-related GO–BP categories (**Supplementary Figure 4**). The gene expression of some of the pro-inflammatory cytokines (e.g., *CCL5* and *CXCL10*) related to hyperinflammation in severe cases of SARS-CoV-2 infection was upregulated in DEG set A (UP) and again downregulated in DEG set B (DOWN) (**Figure 3B**). Similar trends of dysregulation were observed for multiple genes of type III interferons (*IFNL1*, *IFNL2*, and *IFNL3*) and ISGs (*OAS1*, *OAS2*, *OAS3*, *MX1*, and *MX2*) upon comparing both contrasts. Therefore, potential immune modulatory effects could be the consequence of the impairment of virus replication induced by the inhibitor ATR-002. This is also supported by the observation that the majority of the total number of DEGs in contrast B is not associated to immune regulatory processes during gene enrichment analysis (**Figures 4B,C**). A lower viral load may indirectly lead to the reduced expression of immune response genes. Therefore, we cannot assess whether reduced replication may also have a dampening effect in addition to ATR-002 on the expression of innate immune-response-related genes. To separate the infection- and inhibitor-induced effects from each other, MEK1/2 inhibition in a non-infectious environment was further analyzed.

### 3.5 MEK1/2 inhibition in a non-infectious environment induces a shift toward downregulated DEGs in the mRNA expression profile

**Figure 5:**
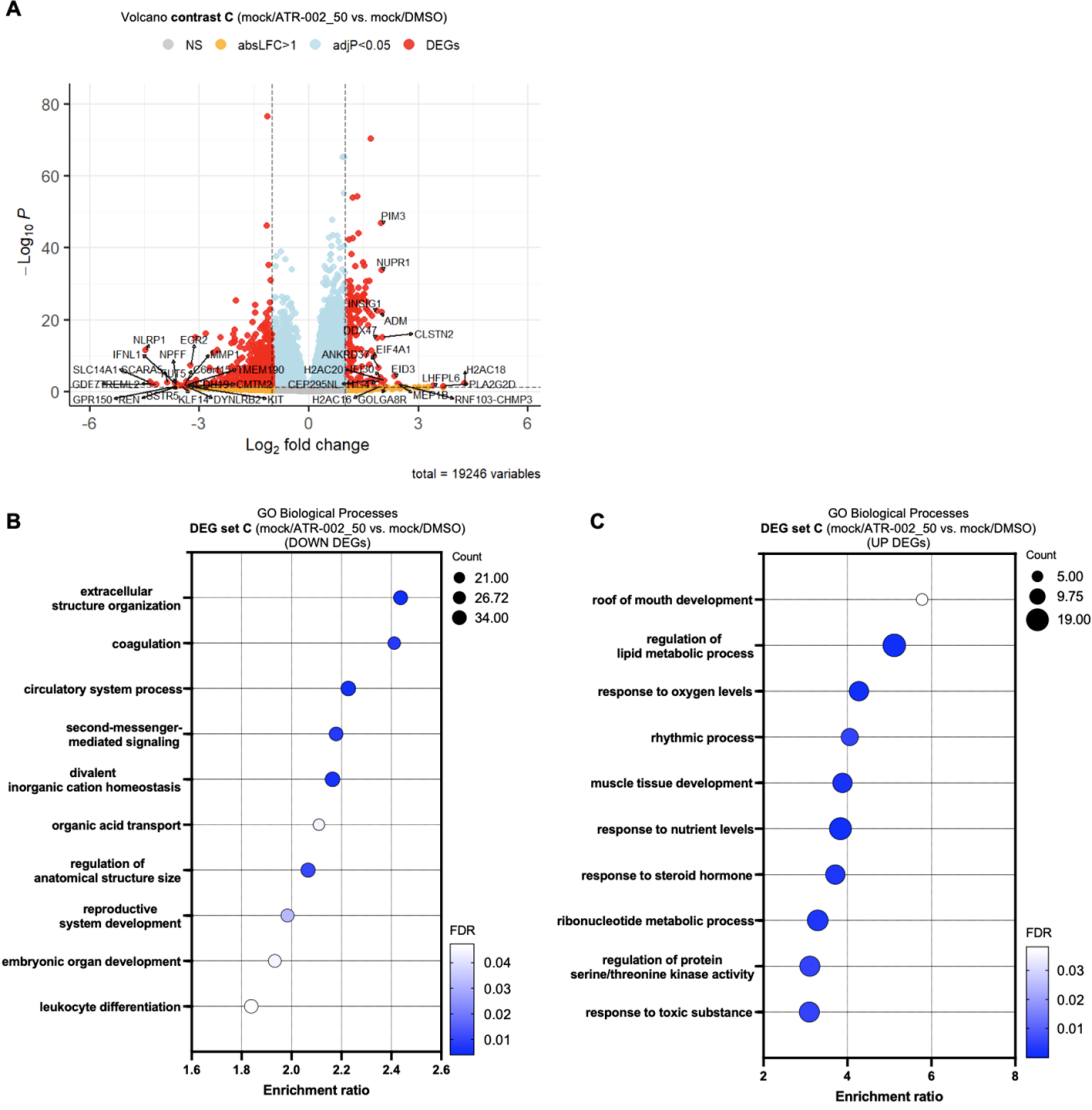
Overview of DEGs in ATR-002 (50 μM)-treated mock-infected Calu-3 cells (contrast C: mock/ATR-002_50 vs. mock/DMSO). **(A)** Volcano plot of contrast C showing all significant DEGs (red) and annotation (gene symbols) of the top 20 differentially expressed genes (up- and downregulated). Genes with adj. *p*-value <0.05 are shown in blue. Genes with an absolute log_2_ fold change >1 are shown in yellow. Log_2_ fold change is plotted on the x-axis and –log_10_ adj. *p*-value is plotted on the y-axis. **(B)** Over-representation analysis (ORA) of DEG set C (DOWN) showing gene ontology terms of biological processes (GO-BPs) of downregulated DEGs. **(C)** Over-representation analysis (ORA) of DEG set C (UP) showing gene ontology terms of biological processes (GO-BPs) of upregulated DEGs. (B/C) The circle size corresponds to the overlapping counts of genes with the respective gene set.

To show the effects of ATR-002 on cellular gene expression in a non-viral environment, especially in prospect of the influence on the intrinsic innate immune response, the expression profile of DMSO-treated control vs. inhibitor-treated cells (contrast C) was elucidated in more detail. The addition of MEK1/2 for 48 h in Calu-3 cells induced a pronounced shift in the mRNA expression profile compared to DMSO-treated cells, which was characterized by a larger number of downregulated mRNA transcripts (**Supplementary Figure 2C**). The distribution of the total dysregulated genes and the top 20 dysregulated genes by the inhibitor treatment is displayed as a volcano plot (**Figure 5A**). Among the significantly (log_2_ fold > 1, adj. *p* < 0.05) DEGs, strongest downregulation was observed for *NLRP1*, *SLC14A1*, and *TREML2* in the DEG set of the 20 most downregulated genes (**Figure 5A**; **Supplementary Table 8**). The three most upregulated genes due to the inhibition of MEK1/2 were *H2AC18*, *PLA2G2D*, and *LHFPL6* (**Supplementary Table 9**). Performing gene enrichment analysis of the DEG set C (DOWN) of 665 downregulated genes revealed not only biological functions of genes primarily associated to extracellular structure organization (GO:0043062), coagulation (GO:0050817), and circulatory system processes (GO:0003013) but also developmental and differentiation processes (e.g., reproductive system development (GO:0061458) or leukocyte differentiation (GO:0002521)) (**Figure 5B**). Considering the function of the upregulated genes (DEG set C (UP)), the GO analysis identified similar processes as in DEG set B (UP) (**Figure 4C**) such as regulation of lipid metabolic process (GO:0019216), *response to oxygen levels* (GO:0070482), response to nutrient levels (GO:0031667), and response to toxic substance (GO:0009636). Direct analysis of hallmark genes of inflammation (**Figure 3B**; **Supplementary Tables 14**, **15**) consisting of cytokines, growth factors, and transcription factors further indicated no direct regulatory connection of the Raf/MEK/ERK signaling cascade with inflammation-associated downstream targets. MEK1/2 inhibition could only downregulate the expression of *CXCL11*, while other genes (e.g., *ADM*, *NFKBIA*) were upregulated in contrast C (**Supplementary Tables 14**, **15**).

Additionally, genes associated with regulation of protein serine/threonine kinase activity (GO:0071900) were affected in DEG set C (UP) due to MEK1/2 inhibition. Interestingly, the gene transcripts of dual-specificity phosphatases (DUSPs) were upregulated, which are responsible for dephosphorylating MAP kinases, like ERK, JNK, and p38 (29, 30) and thereby negatively regulating the kinase activity. An increase of DUSPs upon MEK1/2 inhibition as seen for both DEG sets B and C (UP) could therefore additionally lead to decreased Raf/MEK/ERK pathway activation and contribute to the anti-viral and possibly also to the anti-inflammatory phenotype.

These data demonstrate that MEK1/2 inhibition alone in a non-infectious environment already led to a major shift in mRNA expression and dysregulation of numerous genes. Nevertheless, GO-BP analysis did not directly reveal effects on the mRNA expression levels of genes known to be involved in the innate immune response or direct effect on the expression levels of cytokine transcripts. Therefore, we specifically searched for genes regulated via the Raf/MEK/ERK pathway and MEK1/2 inhibition that may potentially influence innate immunity.

### 3.6 Screening for significantly altered expression of genes associated to immune regulatory processes affected by ATR-002

**Figure 6:**
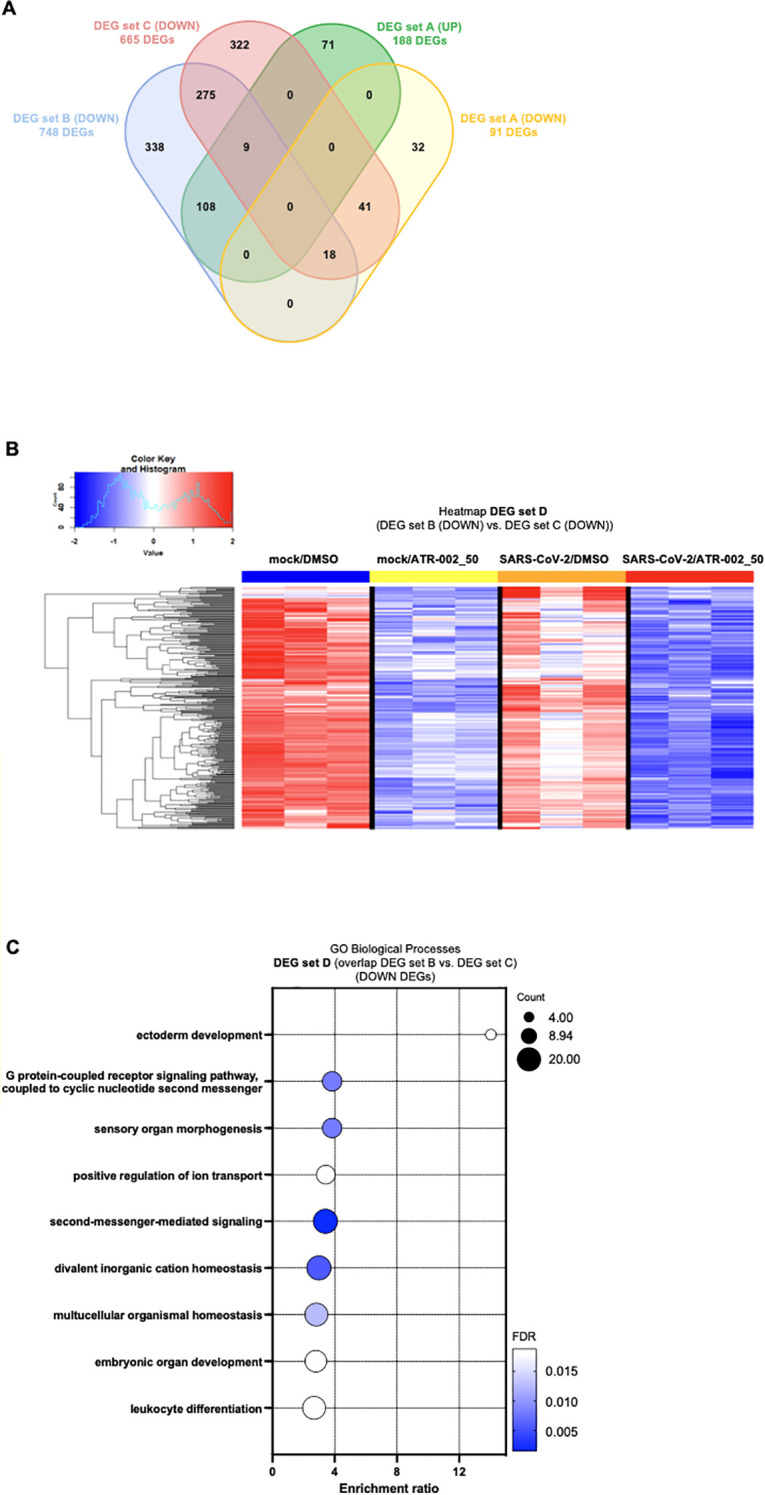
Overview of DEGs in the overlap of DEG set B and DEG set C (DOWN). **(A)** Four-way Venn diagram of DEG set B (DOWN (blue)), DEG set C (DOWN (red)), and DEG set A (UP (green) and DOWN (yellow)), showing the overlapping downregulated DEGs in the ATR-002-treated SARS-CoV-2 infection (contrast B) and the inhibitor treatment alone (contrast C) and which genes were already affected by the simple SARS-CoV-2 infection (contrast A). **(B)** Heatmap of the DEGs identified in DEG set D, showing the relative gene expression levels relative to DMSO-treated samples. **(C)** Over-representation analysis (ORA) of DEG set D (DOWN) showing gene ontology terms of biological processes (GO-BPs) of the overlapping downregulated DEGs. The circle size corresponds to the overlapping counts of genes with the respective gene set.

**Figure 7:**
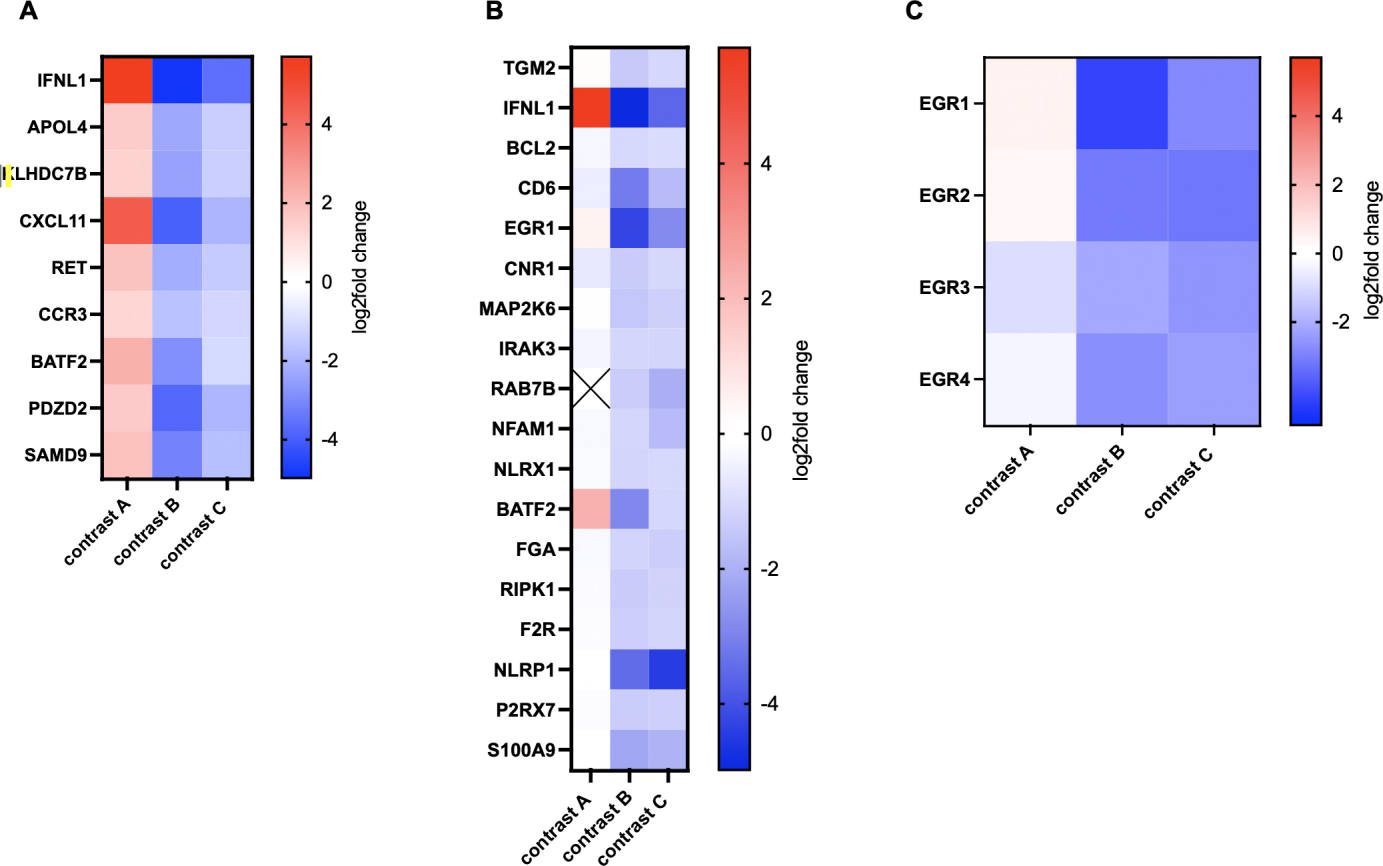
GOIs with differential expression induced by the inhibitor ATR-002. **(A)** Heatmap of overlapping DEGs (DEG set of nine genes from **Figure 6A**), which were deregulated in DEG set B (DOWN), DEG set C (DOWN), and DEG set A (UP). Depicted is the log_2_ fold change from each gene and the respective contrast, enabling comparison of regulation between contrasts. **(B)** Heatmap of the downregulated 18 genes of interest (GOIs) connected to the immune response in DEG sets B and C (DOWN). Genes under the set threshold (log_2_ fold change > 1, adj. *p*-value < 0.05) are indicated as X in the heatmap. **(C)** Heatmap of the four EGR subtypes and their differential expression in the contrasts A–C. Depicted in the heatmaps are the log_2_ fold changes from each gene and the respective contrast, enabling comparison of regulation between contrasts.

A major aim of this study was to identify genes which are dysregulated by the MEK1/2 inhibitor ATR-002 involved in immune regulatory processes. Indeed inhibitor treatment in combination with infection did reveal the dysregulation of genes belonging to such categories, like response to virus or positive regulation of cytokine production. Due to the fact that inhibition of the Raf/MEK/ERK pathway reduces virus replication and thereby decreases the immunostimulatory trigger, this gene subset alone would not allow the identification of specific genes or direct effects on the expression of pro-inflammatory cytokines. To overcome this problem, an overlapping gene set of DEG set B (DOWN) vs. DEG set C (DOWN) was generated to identify genes regulated by ATR-002 independently from infection. At the same time, dysregulation of those genes needed to be observed in the case of infection to ensure relevance for a therapeutic approach in a four-way Venn diagram. Out of the 665 genes downregulated by ATR-002 treatment alone (DEG set C (DOWN)), 302 genes overlapped with DEG set B (DOWN) (inhibitor treatment without infection) (**Figure 6A**; **Supplementary Table 10**). These 302 genes (DEG set D) were specifically downregulated by the inhibitor ATR-002 (**Figure 6B**). Gene enrichment analysis, however, did not clearly show enriched terms for any immune-response-related processes (**Figure 6C**). Nine genes (*IFNL1*, *APOL4*, *KLHDC7B*, *CXCL11*, *RET*, *CCR3*, *BATF2*, *PDZD2*, and *SAMD9*) (**Supplementary Table 11**) of the 302 genes of DEG set D (**Figure 6A**) reappeared in the gene set of DEG set A (UP). Dysregulation of this small DEG set D was visualized as heatmap (**Figure 7A**) to compare infection- and inhibitor-induced effects. Inhibition of the Raf/MEK/ERK pathway decreased the mRNA levels throughout the DEG sets, and log_2_ fold changes decreased even more in the inhibitor-treated SARS-CoV-2 infection (DEG set B (DOWN), **Figure 7A**). This indicates a stronger effect of the inhibitor ATR-002 in infection, which was emphasized by the impaired viral replication. SARS-CoV-2 infection upregulated the expression of those nine transcripts such as *IFNL1* or *CXCL11*, which are described to be involved in processes like ISG production and recruitment of immune cells (31, 32). The downregulation of those genes could be associated with the immune regulatory potential of ATR-002.

The remaining 275 genes out of DEG set D (**Figure 6A**), which were regulated by the inhibitor independently from infection (DEG set C (DOWN)) as well as in the MEK1/2-inhibited SARS-COV-2 infection (DEG set B (DOWN)), were further analyzed with respect to their involvement in immune regulatory processes. To identify more genes of interest, the GO terms from DEG set A (DOWN) response to type I interferon, response to virus, defense response to other organism, positive regulation of defense response, and positive regulation of cytokine production and the accordingly assigned genes were used to find overlapping genes in DEG set D. A total of 18 genes (*TGM2*, *IFNL1*, *BCL2*, *CD6*, *EGR1*, *CNR1*, *MAP2K6*, *IRAK3*, *RAB7B*, *NFAM1*, *NLRX1*, *BATF2*, *FGA*, *RIPK1*, *F2R*, *NLRP1*, *P2RX7*, and *S100A9*) were identified (**Figure 7B**; **Supplementary Table 12**), which were categorized into the GO groups, namely (1), positive regulation of cytokine production (GO:0001819) (2), positive regulation of defense response (GO:0031349), and (3) defense response to other organism (GO:0098542). The strongest dysregulation was observed for *NLRP1* (-4.45939 log_2_ fold change), *IFNL1* (-3.60283 log_2_ fold change), and *EGR1* (-2.81599 log_2_ fold change) in contrast C, though *IFNL1* (-4.97961 log_2_ fold change) and *EGR1* (-4.2894 log_2_ fold change) were even more strongly downregulated in DEG set B (DOWN) (**Figure 7B**). Additionally, SARS-CoV-2 infection and inhibitor treatment also affected the expression levels of other EGR isoforms (EGR2-4) (**Figure 7C**; **Supplementary Table 13**). These targets (*IFNL1* and *NLRP1*) were chosen for further validation and evaluation in terms of their regulation via the Raf/MEK/ERK signaling axis and importance for immune regulatory processes.

Since not only downregulation but also upregulation of transcripts could act on the regulation of inflammatory processes, it was important to elucidate the upregulated gene transcripts upon inhibition of MEK1/2. To identify more GOIs, analyses of upregulated DEG sets B and C (UP) were performed as described above. A total of 83 genes were identified to be upregulated by the inhibitor itself as well as in combination with SARS-CoV-2 infection (**Supplementary Figure 5A**; **Supplementary Table 16**). No GO terms related to immune regulatory processes were shown to be upregulated in this DEG set (**Supplementary Figure 5B**).

### 3.7 Validation of NLRP1 and IFNL1 expression upon MEK1/2 inhibition in SARS-Cov-2 infections

Previous analyses on specific genes which are directly deregulated upon inhibition of MEK1/2 with the inhibitor ATR-002 identified NLRP1 and IFNL1 as potential targets involved in the regulation of innate immunity. To validate that these genes are regulated by the inhibitor during infection and independently from viral stimulation, qRT-PCR was performed after mock and SARS-CoV-2 infection and following inhibitor treatment for these targets. Treatment and infection were performed according to the experimental design for the RNA-seq analysis, additionally including the SARS-CoV-2 omicron B.1.1.529 (BE.1.1) variant.

**Figure 8:**
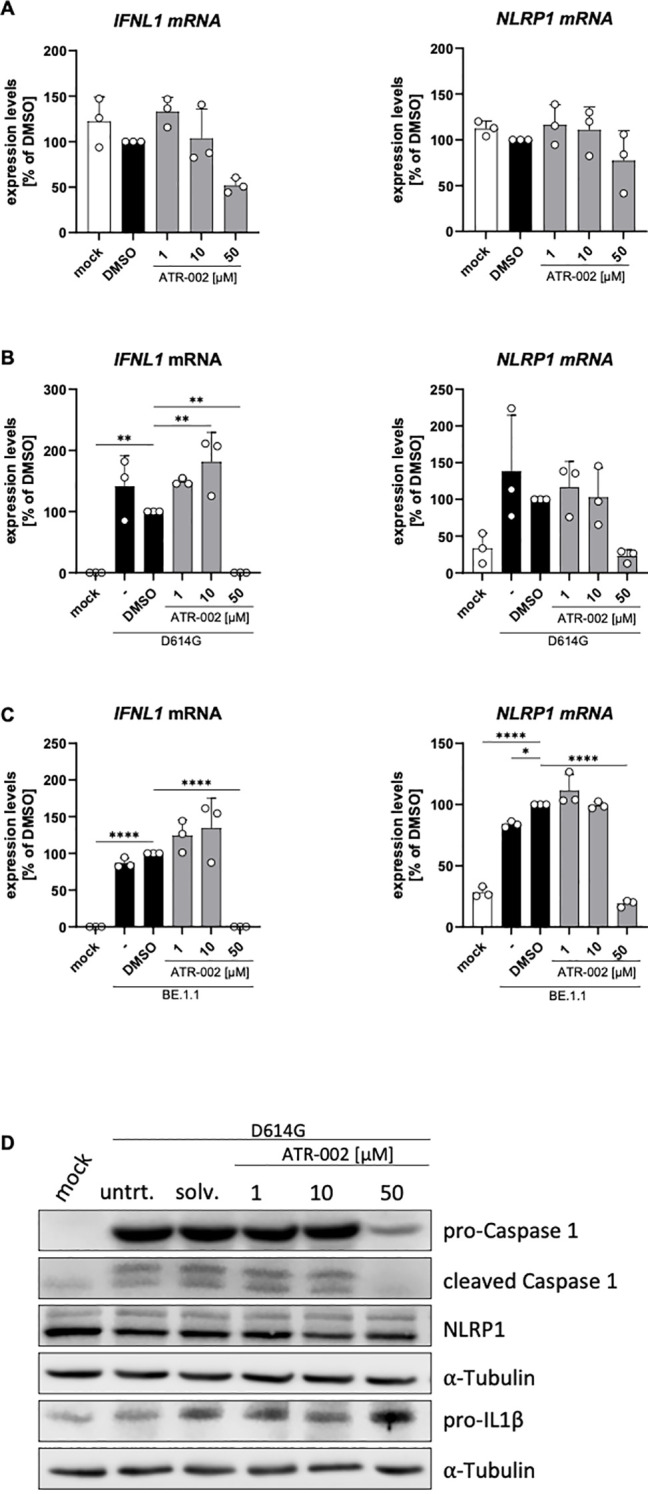
Validation of mRNA and protein expression of GOIs. **(A)** mRNA expression levels of mock-infected and ATR-002-treated Calu-3 cells 48 h.p.i. **(B)** mRNA expression levels of SARS-CoV-2 (D614G)- and **(C)** SARS-CoV-2 (BE.1.1)-infected (MOI 0.01) and ATR-002 (1, 10, and 50 µM)-treated Calu-3 cells at 48 h.p.i. The mRNA levels were determined by qRT-PCR, and *n*-fold expression was normalized to DMSO (arbitrarily set to 100%) ±SD. The data represent three biological replicates and passed ordinary one-way ANOVA followed by Dunnett’s multiple comparison (*p ≤ 0.05, **p ≤ 0.01, ****p ≤ 0.0001). **(D)** Protein expression analysis of SARS-CoV-2 (D614G)-infected (MOI 0.01) and ATR-002 (1, 10, and 50 µM)-treated Calu-3 cells via western blot of pro-caspase 1, cleaved caspase 1, NLRP1, pro-IL1β, and tubulin. The data show one representative replicate out of three biological replicates.

Basal mRNA transcription was detectable for *IFNL1* and *NLRP1* (**Figure 8A**), which could be modulated by the inhibitor ATR-002 at the concentration of 50 µM, proving the negative regulatory potential of the compound. Moreover, gene transcription was further evaluated in the context of SARS-CoV-2 infection using the D614G (**Figure 8B**) and the Omicron B.1.1.529 (BE.1.1) (**Figure 8C**) variants. While infection with both SARS-CoV-2 variants increased the induction of *IFNL1* and *NLRP1* expression, treatment with the MEK1/2 inhibitor caused a significant reduction in mRNA levels to the level of basal expression for both virus variants and targets (**Figures 8B,C**). Lower concentrations of ATR-002 could not influence the gene expression of *NLRP1* in both infections. In contrast, *IFNL1* mRNA levels were slightly increased at these concentration (1 and 10 µM), hinting toward a supporting mechanism of the anti-viral response induced by MEK1/2 inhibition at low doses. Overall, this proved as an independent mode of action of ATR-002 from the virus variants. In combination with the effects seen for MEK1/2 inhibition of uninfected cells, it was hypothesized that MEK1/2 inhibition could directly regulate the gene expression of these genes.

To further analyze the potential influence of the Raf/MEK/ERK signaling cascade on innate immunity, NLRP1 protein expression and the corresponding processing of pro-Caspase 1 and pro-IL1β were investigated by western blot analysis (**Figure 8D**). As proof of principle, the D614G variant was used for the infection, and samples were analyzed 48 h.p.i. While NLRP1 protein expression was not induced by SARS-CoV-2 infection and not affected by the inhibitor treatment, reduction of the cleaved caspase-1 subunit p20 was visible upon ATR-002 treatment. Furthermore, increased protein levels of pro-IL1β were observed upon MEK1/2 inhibition, which proved the loss of caspase-1 activity upon pathway impairment. Taken together, ATR-002 possesses the potential to regulate the activity of caspase-1 and thereby influences the processing of the pro-inflammatory cytokine IL1β.

## 4 Discussion

The inhibition of the Raf/MEK/ERK pathway by MEK1/2 inhibitors, e.g., ATR-002, CI-1040, trametinib, or U0126, leads to a reduction in the viral replication of multiple viruses (e.g., SARS-CoV-2, influenza virus, and RSV) (17, 18, 20, 21, 33). These host-targeted antiviral acting drugs block the Raf/MEK/ERK cascade at its bottleneck MEK1/2 and thereby disrupts the intracellular signaling network and interactions between distinctive pathways. This potentially impacts diverse cellular responses in a viral infection, such as the expression of pro-inflammatory cytokines as hypothesized in multiple studies (17, 21, 34).

SARS-CoV-2 infection (contrast A) induced a clear innate immune gene expression response with upregulation of transcripts of multiple pro-inflammatory cytokines as well as anti-viral genes. Interestingly, MEK1/2 inhibition, upon viral infection, led to the reversal of this induced expression, affecting 117 genes mostly associated with inflammatory and anti-viral functions. Since inhibitor treatment of the SARS-CoV-2 infection reduced the virus RNA accumulation resulting from decreased viral replication, also shown by Scheiber et al. (2022), the dampened immune response observed could be an indirect consequence. Other studies indicate a more direct regulatory function of the Raf/MEK/ERK pathway on innate immune regulatory pathways apart from the reduced viral load upon MEK1/2 inhibition. It was shown that even in “simulated infections” using the pathogen-associated molecular patterns (PAMP) vRNA or polyI:C, MEK1/2 inhibition led to reduced expression levels of selected pro-inflammatory cytokines (17, 21). Additionally, further immune regulatory functions of the Raf/MEK/ERK downstream targeting RSK1 and RSK2 were observed in multiple studies. Both isoforms were identified to modulate the activation of NF-κB and thereby act on the expression of cytokines (35, 36). Dysregulation of the pathway influences the kinase activity of ERK and downstream targets (e.g., RSK) responsible for the regulation of immune regulatory pathways, resulting in the activation of different transcription factors such as NF-κB and IRF. As this study was designed to generate a transcriptional profile of the impact of MEK1/2 inhibition during SARS-CoV-2 infection of cells, implications on the activity of intracellular signaling were not within the scope of this study and not depicted here. While the MAPK/ERK pathway, as described previously, could intersect the activation of the NF-κB complex by direct protein–protein interactions, transcriptional regulation of other transcription factors relevant for innate immunity could be mediated by the Raf/MEK/ERK signaling cascade as well. Considering the regulation of transcription factors and their importance for innate immunity, the complexity of the finely tuned immune response was demonstrated once again. A specific analysis of hallmark genes for inflammatory response revealed higher expression levels of the *NF*κ*BIA* gene (**Supplementary Tables 14**, **15**), also known as IκBα, when MEK1/2 is impaired. Increased levels of the NFκB inhibitor IκBα could negatively affect the inflammatory response and suppress the expression of pro-inflammatory factors (37). Considering other hallmark genes for inflammation (Molecular Signatures Database (MSigDB)) for the analysis of MEK1/2-inhibitor-regulated gene expression and its influence on immunity, infection caused significant upregulation of some of these genes (**Supplementary Tables 14**, **15**), which was decreased by the inhibitor treatment during infection, while ATR-002 treatment without the viral stimulus had no clear effect, possibly due to the reduced viral replication. Therefore, direct gene regulation of these specific genes via MEK1/2 inhibition seems unlikely, though it should be considered that we are missing any kind of immune trigger in this specific contrast C. Therefore, steady-state levels of pro-inflammatory cytokines could not be modulated. In this study, more specifically in contrast B, we were limited in our interpretation by the reduced viral replication upon MEK1/2 inhibition. To overcome this problem but still activate the immune response and the signaling cascade, the approach of virus-mimicking stimuli, like inactivated viruses or treatment with PAMPs, would be a future approach. This strategy would allow the induction of inflammatory genes as well as the activation of the Raf/MEK/ERK pathway without affecting viral replication. Even though we were facing some limitations in this study, it was possible to screen for factors which play a role in innate immunity and can be regulated by the Raf/MEK/ERK pathway.

The regulation of antiviral mechanisms is a multi-dimensional system that comprises a network of intracellular signaling cascades, which “communicate” and impact the expression of pro- and anti-inflammatory factors. The Raf/MEK/ERK signaling cascade may not exert its primary influence on innate immunity through direct regulation of cytokine expression via direct gene targeting but rather contributes more broadly by orchestrating immune-related mechanisms and pathways, potentially inducing the transcription of key regulatory factors that modulate cytokine production in either an activating or repressing manner. To identify such factors, which are regulated by impairment of the Raf/MEK/ERK signaling cascade, it was focused on a data set centered on the inhibitor alone (contrast C). Because of the major issue that interference with viral replication could impact the immune response, virus-related effects could hardly be mistaken for inhibitor-induced effects on the immune response. Therefore, the valuable information found in contrast C was used to compare these DEGs with DEGs affected by inhibitor treatment in SARS-CoV-2 infection. A comparison of these two contrasts was performed to narrow down the list of GOIs and to ensure potential effects during infection as well. This resulted in 302 overlapping genes (DEG set D). Additionally, this screening allowed to search for transcripts related to immune regulatory processes, of which 18 genes were associated to such processes. Nucleotide-binding domain, leucin-rich repeat protein 1 (*NLRP1*) was the transcript most strongly downregulated upon MEK1/2 inhibition. This gene is involved in the formation of the NLRP1 inflammasome protein complex, which is responsible for the cleavage and activation of the cytokines IL1β and IL18, e.g., upon viral sensing (38, 39). Activation or transcription of the NLRP1 inflammasome is initiated via ERK-mediated activation of the transcription factor ATF4, a downstream effector of the Raf/MEK/ERK pathway (40). This connects the observed downregulation of the transcript upon MEK1/2 inhibition with Raf/MEK/ERK signaling pathway and its potential to act as a target for ant-inflammatory treatment. The following validation confirmed the MEK1/2-mediated regulation of NLRP1 on the mRNA level. While the protein levels were not affected by the treatment, downstream effectors were shown to be less activated, which resulted in increased pro-IL1β levels. Interestingly, the efficacy of the inhibitor on mRNA expression did not depend on the SARS-CoV-2 variants used for the infection. Older variants of concern (Delta) or newer variants (Omicron) associated with less severe disease did respond similarly to the regulation of gene expression of NLRP1 as well as IFNL1. This emphasizes on the broad mode of action of the inhibitor ATR-002, which could be widely used in SARS-CoV-2 infections relying on the Raf/MEK/ERK cascade.

IFNL1, as a family member of type III interferons, was highly upregulated during SARS-CoV-2 infection as described in different studies (41, 42), thriving the antiviral response. MEK1/2 inhibition reversed this upregulation in SARS-CoV-2 infection in this study, suggesting a reduction in the antiviral response induced by the inhibitor and decreased viral progeny. However, DEG set D revealed that no other interferons or ISGs were downregulated specifically by the inhibitor besides IFNL1. This indicates no impairment of the antiviral response in the SARS-CoV-2 infection due to ATR-002, which was already postulated in previous studies (17, 21). Moreover, we identified *IFNL1* as a potential new direct target gene of the Raf/MEK/ERK signaling axis, which has not been demonstrated before. For future perspectives, it might be interesting to investigate the exact underlying gene regulatory mechanism.

The well-characterized C2H2 zinc finger transcription factor, early growth response 1 (EGR1), here shown to be downregulated upon inhibition of MEK1/2 independent from infection, as well as under stimulation with SARS-CoV-2, is described to be directly transcriptionally regulated via ERK1/2-dependent ELK1 phosphorylation. A downstream target of the signaling cascade binds to serum response elements (SRE) and thereby targets gene activation (43–45). EGR1 is known to act as a transcriptional regulator for several genes, such as multiple cytokines, like IL6, IL8, CCL2, and TNFα (46, 47). Regulation of gene expression of *EGR1* could therefore impact the expression of pro-inflammatory cytokines in an ERK/ELK1-dependent manner. However, we were not able to further validate EGR1 expression upon SARS-CoV-2 infection and ATR-002 treatment. Zhao and colleagues (2023) identified EGR1 as a potential target for immune evasion processes resulting in decreased expression mediated via the viral N protein (48). Less sensitive methods in contrast to RNA-seq analysis, such as qRT-PCR and western blot, used for validation could therefore not detect the mRNA or protein expression of this target. According to previously published data on the functionality of EGR1 in SARS-CoV-2 infections, virus-mediated suppression of EGR1 could be an immune evasion strategy of the virus, emphasizing on its importance for immunity.

In conclusion, we showed that MEK1/2 inhibition by ATR-002 did lower the expression of antiviral factors such as type I interferons and ISGs as well as inflammatory genes, which were activated by SARS-CoV-2 infection. Moreover, high gene regulatory potential of the inhibitor ATR-002 was observed, which emphasized on its potential to modulate diverse molecular processes (e.g., immune response). Screening for factors which can be regulated by the Raf/MEK/ERK pathway and possess the potential to modulate innate immunity revealed the transcription factor EGR1 as a possible candidate. Moreover, regulation of NLRP1 could influence the processing of pro-IL1β via the reduced activity of caspase 1. Its direct ERK1/2-dependent transcriptional regulation and previously described involvement in immune regulatory processes identified this factor for the first time as an interesting modulator of virus-induced innate immune responses. Nevertheless, it is most unlikely that these factors harbor the potential all alone to regulate inflammatory gene expression. To unravel the mechanism behind the dysregulation of cytokines upon usage of a MEK1/2 inhibitor, it is necessary to think of a more complex intracellular interplay and look at multiple levels of potential interactions. In this case, it will be important to also consider the dynamics of viral infections as well as host responses. As attempted in this study to separate virus-induced effects from inhibitor-induced effects, this should be considered for future analyses using a virus-mimicking system (e.g., inactivated virus and vRNA).

## Data Availability

Raw sequence reads and the normalized, expression matrix are available at the GEO public database (https://www.ncbi.nlm.nih.gov/geo/), ID: GSE303020.
