# Supplementary material for: MEK1/2 inhibitor ATR-002 reshapes host transcriptome and modulates immune regulatory genes in SARS-CoV-2 infection
Source: Front Immunol. 2026 May 29;17:1724353. doi: 10.3389/fimmu.2026.1724353 (PMC13260356; doi:10.3389/fimmu.2026.1724353)
Supplement: Supplementary file 1 [file DataSheet1.pdf]

Supplementary Material

1 Supplementary Figures and Tables

1.1 Supplementary Figures

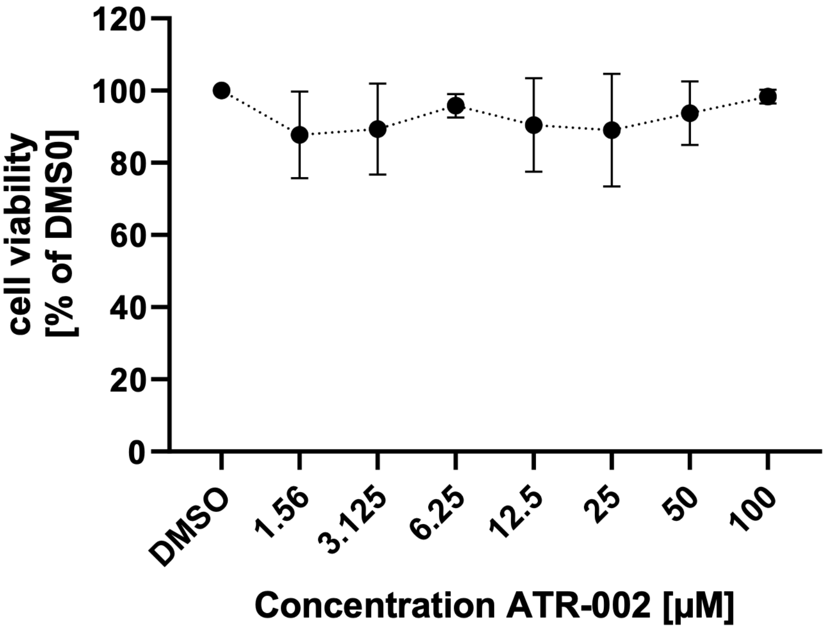

Supplementary Figure 1. Lactate-dehydrogenase (LDH) cell viability assay of ATR-002 treated Calu-3 cells.

Calu-3 cells were treated with the indicated concentrations of the inhibitor ATR-002 for 48 h. Cell viability was measured via LDH release and activity. Two independent replicates are depicted. DMSo was arbirtraily set to 100 %.

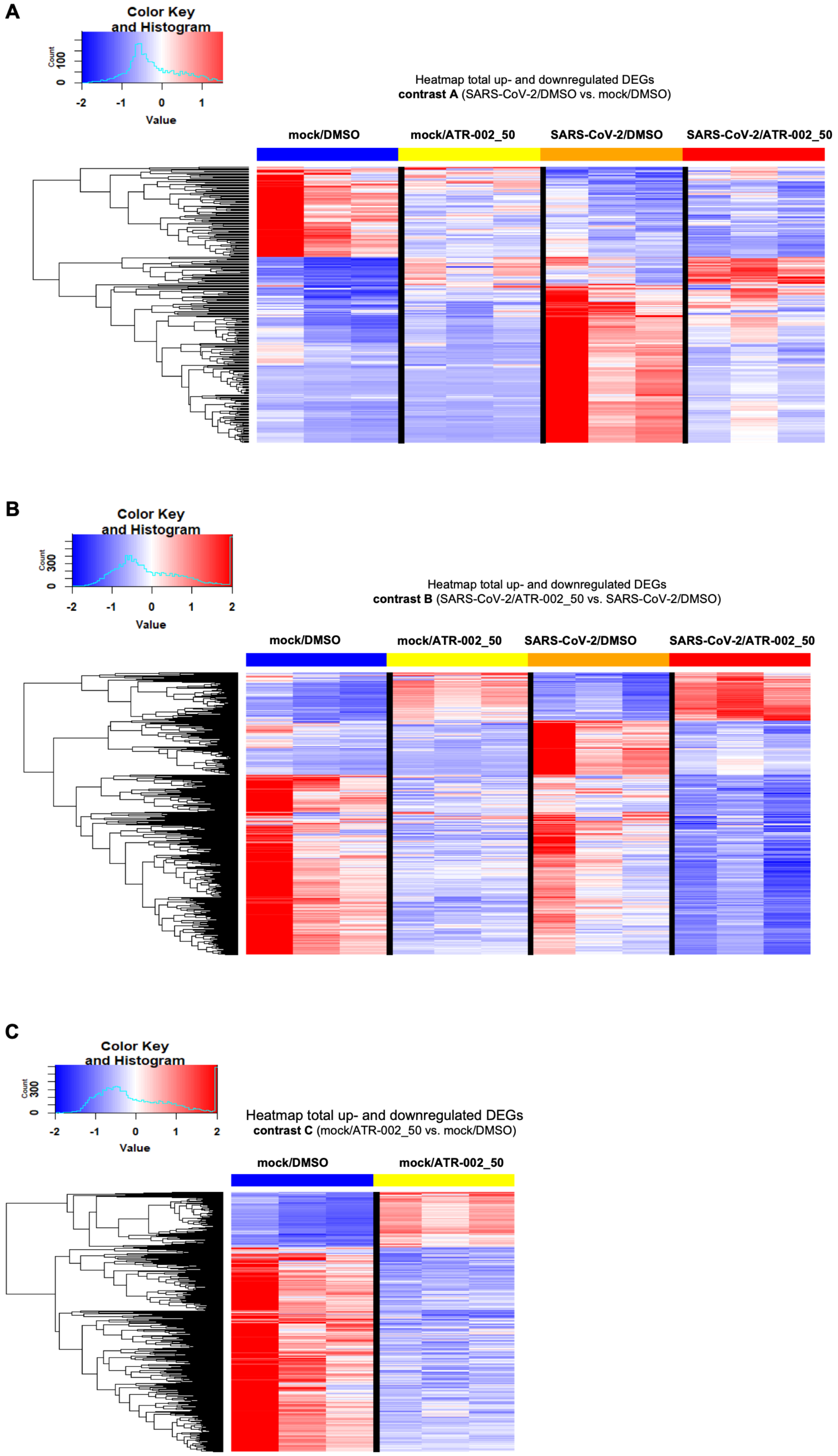
A
Color Key and Histogram
Count
0
100
-2
-1
0
1
Value
Heatmap total up- and downregulated DEGs
contrast A (SARS-CoV-2/DMSO vs. mock/DMSO)
mock/DMSO
mock/ATR-002_50
SARS-CoV-2/DMSO
SARS-CoV-2/ATR-002_50
B
Color Key and Histogram
Count
0
300
-2
-1
0
1
2
Value
Heatmap total up- and downregulated DEGs
contrast B (SARS-CoV-2/ATR-002_50 vs. SARS-CoV-2/DMSO)
mock/DMSO
mock/ATR-002_50
SARS-CoV-2/DMSO
SARS-CoV-2/ATR-002_50
C
Color Key and Histogram
Count
0
300
-2
-1
0
1
2
Value
Heatmap total up- and downregulated DEGs
contrast C (mock/ATR-002_50 vs. mock/DMSO)
mock/DMSO
mock/ATR-002_50

Supplementary Figure 2. Representation of DEGs in contrast A, B and C.

(**A**) Heatmap of total up- and downregulated DEGs of contrast B showing the relative gene expression levels of SARS-CoV-2 infected Calu-3 cells in contrast to mock/DMSO treated samples. (**B**) Heatmap of total up- and downregulated DEGs of contrast B showing the relative gene expression levels of SARS-CoV-2 infected Calu-3 cells in contrast to mock/DMSO treated samples. (**C**) Heatmap of total up- and downregulated DEGs of contrast C showing the relative gene expression levels of ATR-002 (50 µM) mock infected Calu-3 cells in contrast to mock/DMSO treated samples.

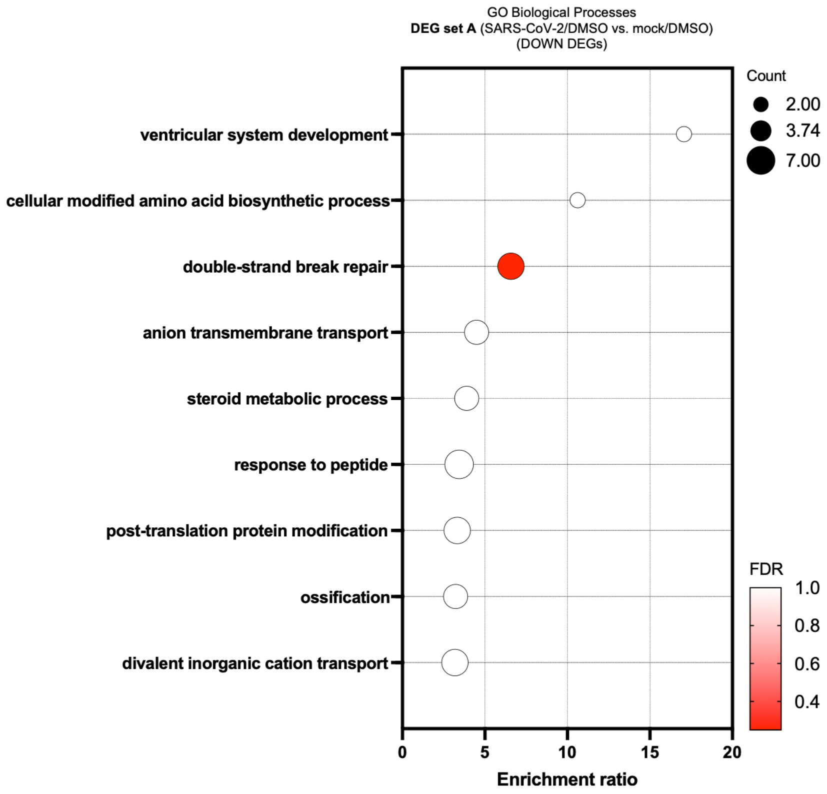

Supplementary Figure 3. Over-representation analysis of DEG set A (DOWN)

ORA of downregulated DEGs of DEG set A showing gene ontology terms of biological processes. Circle size corresponds to the overlapping counts of genes with the respective gene set. False discovery rate (FDR) for the enriched terms was below the set threshold of FDR ≤ 0.05.

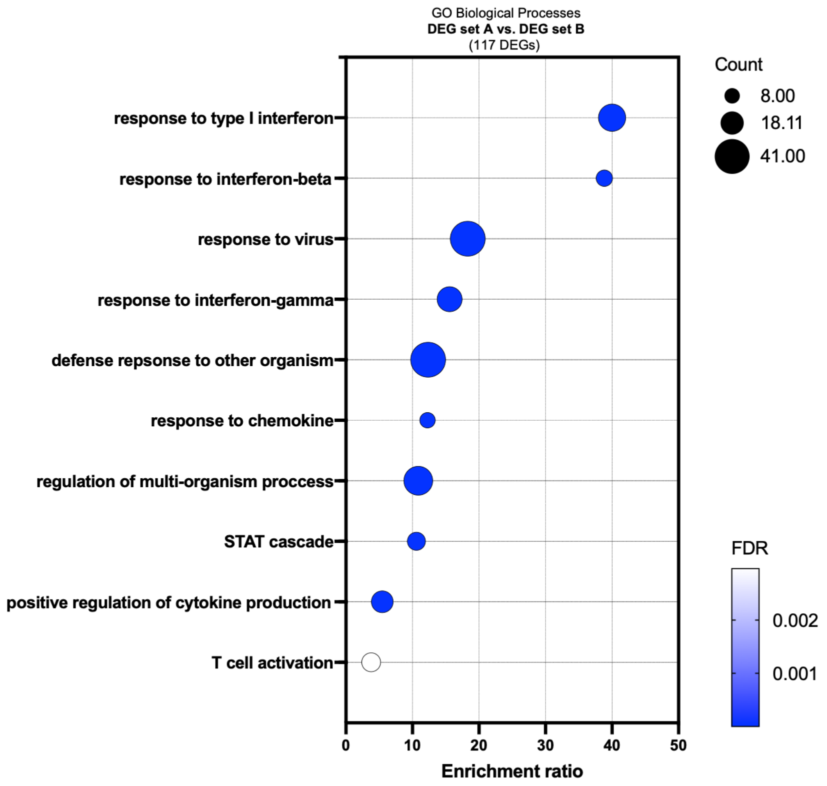

Supplementary Figure 4. Over-representation analysis of overlap between DEG set A (UP) and DEG set B (DOWN)

117 genes were identified in the overlap of the DEG sets. ORA of downregulated DEGs of the performed overlap of DEG set A (UP) and DEG set B (DOWN) showing gene ontology terms of biological processes. Circle size corresponds to the overlapping counts of genes with the respective gene set. Threshold for the false discovery rate for the enriched terms was set to FDR ≤ 0.05.

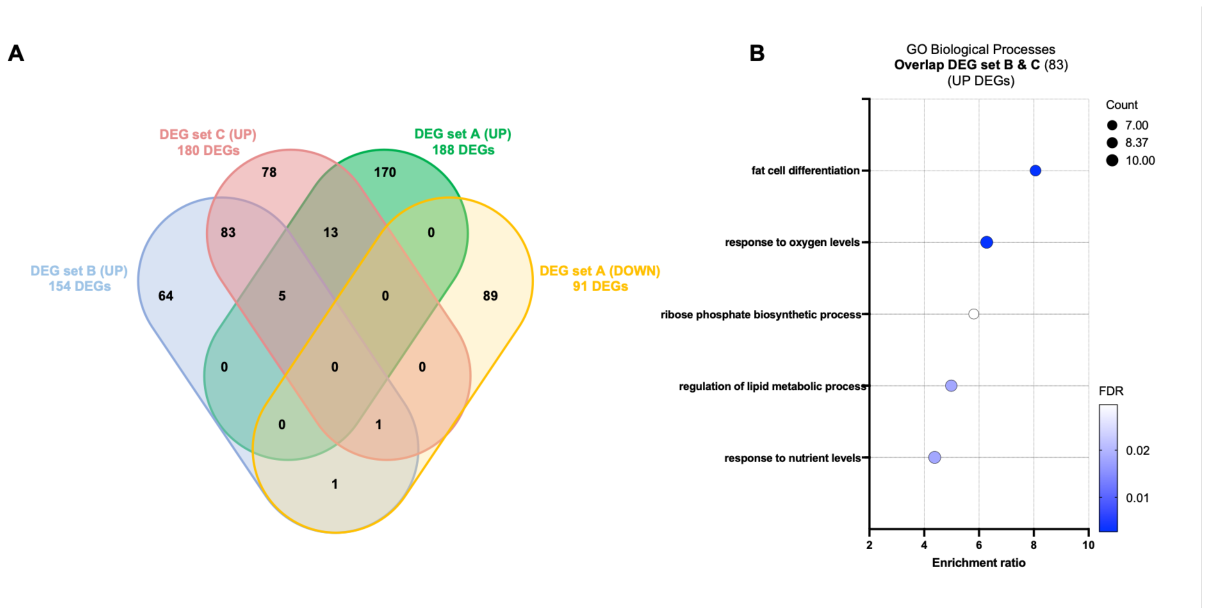

Supplementary Figure 5. Comparison of contrast A, B and C

(**A**) 4-way Venn diagram of DEG set B (UP (blue)), DEG set C (UP (red)) and DEG set A (UP (green) and DOWN (yellow)), showing the overlapping upregulated genes in the ZMN treated SARS-CoV-2 infection (contrast B) and the inhibitor treatment alone (contrast C) and which genes are already affected by the simple SARS-CoV-2 infection (contrast A). (**B**) ORA of upregulated 83 DEGs overlapping in DEG set B and C (UP) showing gene ontology terms of biological processes. Circle size corresponds to the overlapping counts of genes with the respective gene set.

1.2 Supplementary Tables

Table S1 Number of DEGs in the different contrasts.

File Name: Supplementary_Tab_SARS-CoV-2_transcriptome

Description of data: see name of table

Table S2 Upregulated DEGs in contrast A.

File Name: Supplementary_Tab_SARS-CoV-2_transcriptome

Description of data: see name of table

Table S3 Downregulated DEGs in contrast A.

File Name: Supplementary_Tab_SARS-CoV-2_transcriptome

Description of data: see name of table

Table S4 Downregulated DEGs in contrast B.

File Name: Supplementary_Tab_SARS-CoV-2_transcriptome

Description of data: see name of table

Table S5 Upregulated DEGs in contrast B.

File Name: Supplementary_Tab_SARS-CoV-2_transcriptome

Description of data: see name of table

Table S6 Gene list: 117 overlapping genes in contrast A (UP) and contrast B (DOWN).

File Name: Supplementary_Tab_SARS-CoV-2_transcriptome

Description of data: List of gene symbols of the overlapping 117 genes found in the comparison of contrast A (UP) and contrast B (DOWN), showing those genes, which are specifically downregulated in the inhibitor treated SARS-CoV-2 infection. Additionally, those gene symbols of the unique genes found for each contrast are listed.

Table S7 Downregulated DEGs in contrast C.

File Name: Supplementary_Tab_SARS-CoV-2_transcriptome

Description of data: see name of table

Table S8 Upregulated DEGs in contrast C.

File Name: Supplementary_Tab_SARS-CoV-2_transcriptome

Description of data: see name of table

Table S9 Gene list: 302 overlapping genes in contrast B (DOWN) and contrast C (DOWN)

File Name: Supplementary_Tab_SARS-CoV-2_transcriptome

Description of data: List of gene symbols of the overlapping 302 genes found in the comparison of contrast B (DOWN) and contrast C (DOWN), showing those genes specifically downregulated by the inhibitor ZMN. Additionally, those gene symbols of the unique genes found for each contrast are listed.

Table S10 Gene list: Overlap of 9 genes in contrast A, B and C.

File Name: Supplementary_Tab_SARS-CoV-2_transcriptome

Description of data: List of gene symbols of the overlapping 9 genes found in the comparison of contrast A (UP), contrast B (DOWN) and contrast C (DOWN). Shown are those genes, which are upregulated in the SARS-CoV-2 infection and again downregulated by ZMN in the infection and individual inhibitor treatment, indicating an inhibitor specific effect on this gene set.

Table S11 Gene list: GOIs found in DEG set D with immune response related functions.

File Name: Supplementary_Tab_SARS-CoV-2_transcriptome

Description of data: List of gene symbols of the overlapping 18 genes found in the comparison of contrast B (DOWN) and contrast C (DOWN). Shown are those genes, which are specifically regulated by the inhibitor ZMN and could be categorized into GO-BP groups related to the immune response.

Table S12 $\log_2$ fold expression of EGR isoforms in contrast A, B and C.

File Name: Supplementary_Tab_SARS-CoV-2_transcriptome

Description of data: see name of table

Table S13 $\log_2$ fold expression of hallmark cytokines in contrast A, B and C.

File Name: Supplementary_Tab_SARS-CoV-2_transcriptome

Description of data: see name of table

Table S14 $\log_2$ fold expression of hallmark transcription factors in contrast A, B and C.

File Name: Supplementary_Tab_SARS-CoV-2_transcriptome

Description of data: see name of table

Table S15 $\log_2$ fold expression of AP-1 transcription factor subunits in contrast A, B and C.

File Name: Supplementary_Tab_SARS-CoV-2_transcriptome

Description of data: see name of table

Table S16 Gene list: Overlap of contrast A, B and C (UP)

File Name: Supplementary_Tab_SARS-CoV-2_transcriptome

Description of data: List of the gen symbols of the overlapping genes revealed by an 4-way comparison of contrast A (UP), contrast A (DOWN), contrast B (UP) and contrast C (UP).
